# Identification and validation of an immune-associated RNA-binding proteins signature to predict clinical outcomes and therapeutic responses in colon cancer patients

**DOI:** 10.1186/s12957-021-02411-2

**Published:** 2021-10-26

**Authors:** Di Sun, Kui-Sheng Yang, Jian-Liang Chen, Zheng-bing Wang

**Affiliations:** 1 Department of Gastrointestinal Surgery, Affiliated Hospital of Yangzhou University, Yangzhou, 225100 People’s Republic of China; 2 Department of General Surgery, People’s Hospital of Jingjiang, Yangzhou University Medical Academy, Yangzhou, China

**Keywords:** Colon cancer, RNA-binding protein, Immune microenvironment, Prognosis model

## Abstract

**Background:**

The immune infiltration of patients with colon cancer (CC) is closely associated with RNA-binding proteins (RBPs). However, immune-associated RBPs (IARBPs) in CC remain unexplored.

**Methods:**

The data were downloaded from The Cancer Genome Atlas (TCGA) and the patients were divided into four immune subgroups by single sample gene set enrichment analysis (ssGSEA), in which weighted gene correlation network analysis (WGCNA) identified modules of co-expressed genes correlated with immune infiltration. Univariate (UCR) and multivariate Cox regression (MCR) analyses were applied to screen survival-associated IARBPs. Then, a prognostic signature was performed on TCGA dataset. Risk model was constructed based on the TCGA dataset. Based on the median risk score, CC patients were subdivided into low- and high-risk groups. Furthermore, the accuracy and prognostic value of this signature were validated by using Kaplan-Meier (K-M) curve, receiver operating characteristic (ROC). We further validated the findings in Gene Expression Omnibus (GEO) database. Finally, we evaluated the association between gene expression level and drug sensitivity.

**Results:**

Based on the infiltration of immune cells, the TCGA patients were divided into four subgroups. In total, we identified 25 IARBPs, after differential expression and WGCNA analysis. Subsequently, two IARBP signatures (FBXO17 and PPARGC1A) were identified to be significantly associated with the overall survival (OS) of CC patients. K-M survival analysis revealed that the low-risk group correlated with prolonged OS. The prognostic signature was an independent prognostic factor and reflects the immune status of CC patients. Finally, FBXO17 was related with drug sensitivity of bleomycin, gemcitabine, and lenvatinib. PPARGC1A was related to drug sensitivity of dabrafenib, vemurafenib, and trametinib.

**Conclusion:**

A novel two immune-associated RBPs that was established that may be useful in predicting survival and individualized treatment.

**Supplementary Information:**

The online version contains supplementary material available at 10.1186/s12957-021-02411-2.

## Introduction

Colon cancer (CC), one of the most common digestive malignant tumors, has become an important public health issue. Its ranks fourth in incidence and second as a cause of mortality among 36 cancer types worldwide [1]. The incidence of CC is high incidence in the Western world and developed Asian countries [2]. Early detection and treatment have decreased morbidity and mortality. Numerous biomarkers have been revealed in CC [3, 4]. Unfortunately, recurrence or metastasis will occur in approximately 30–50% of patients within 5 years after treatment [5]. The effective prediction model can play a significant role for the accurate assessment of patients’ prognosis and to optimize clinical treatment strategies [6–10]. Recently, a growing number of evidences indicate that the potential effect of the tumor immune microenvironment (TIM) have great potential for predicting the efficacy of immunotherapy as well as prognosis [11]. TIM is composed by immune cells. Adaptive anti-tumor immune responses have been correlated with tumor progression in various cancers, including CC, and tumor development is also dependent on the infiltration of immune cells [12].

RNA-binding protein (RBP) plays a significant role in tumor progression via post-transcriptional modification [13]. Specifically, RNA-binding proteins (RBPs) play essential roles in RNA life, including pre-mRNA processing, modification, localization, RNA stability, and translation [14]. The inflammatory response has been reported to be modulated by modulating mRNA pools in both immune and nonimmune cells [15]. Considering that RBPs can regulate the infiltration degrees of immune cells, whether there are immune-associated RBPs that can be used to accurately evaluate the tumor progression and prognosis of CC patients has not yet been considered.

In the present study, we evaluated the association between the expression of immune-associated RBPs (IARBPs) and prognosis in CC patients. To this end, we train a model to predict the OS of patients with CC.

## Materials and methods

### Data processing

The RNA-Seq data and clinical information of male CC patients were obtained from The Cancer Genome Atlas (TCGA) (398 tumor samples and 39 normal samples) and Gene Expression Omnibus (GEO) (GSE40967) database. The gene expression profile of 580 CC (GSE40967) based on the GPL570 (Affymetrix Human Genome U133 Plus 2.0 Array) platform was also downloaded. A total of 1542 RBPs were obtained from a previous study [16].

### Identification of CC subtypes based on ssGSEA score and differentially expressed genes identification

For each CC dataset, 27 immune cell types were determined using the single sample gene set enrichment analysis (ssGSEA) software implemented in the R GSVA package. Consensus clustering was performed using the “ConsensusClusterPlus” package in R to identify subgroups based on ssGSEA scores. In brief, *k*-means clustering was used, with 50 iterations (each using 80% of the samples). The best-fit number of clusters was determined by the cumulative distribution function (CDF) curve and the changes in the area under the CDF curve. The stromal score, immune score, tumor purity, and estimate score of each included sample was calculated by ESTIMATE algorithm [17]. The CIBERSORT deconvolution algorithm (https://cibersort.stanford.edu/ assessed on 28 December 2020) was used to verify that the infiltration of immune cells from these four immune subtypes were different [18]. Differentially expressed genes (DEGs) between Cluster1 and Cluster2 were determined using the R package Limma. Genes with *P* < 0.05, and [logFoldChange (logFC)] > 1 were considered DEGs. Using the gplots and heatmap in the edgeR package, volcano plots and heat maps of DEGs were constructed.

### Weighted gene co-expression network analysis

DEGs were used to construct a weight co-expression network using the R package “WGCNA” [19]. The topological overlap measure (TOM) was clustered, and gene modules were identified. We calculated the module eigengene (ME) of each module, which represents the expression level for each module. The threshold was set as *P* < 0.05.

### Function and pathway analysis

Gene Ontology enrichment analyses of module genes performed using the Metascape (https://metascape.org/), a gene annotation and analysis resource.

### Building a prognostic model

Univariate Cox regression (UCR) analysis was performed to screen out which RBP-related genes associated with the OS of patients. Then, the multivariate Cox regression (MCR) test for the coefficients (bi) of the hub genes was performed. This model was developed as Risk score = ∑i = 1N(Exp(i) · coe(i)). Based on the median risk score, the patients were divided into low-risk or high-risk groups. Then, the ROC curve was conducted to evaluate the predictive accuracy. The K–M survival curve was done to assess whether there was a survival difference between 2 categories. To validate the result, we implemented the same procedure on the validation cohort.

### Drug sensitivity correlation analysis

The drug sensitivity was downloaded from the CellMiner database (https://discover.nci.nih.gov/cellminer/) [20, 21]. R package “impute”, “limma”, “ggplot2”, and “ggpubr” were used for the data processing, statistical analysis, and result visualization.

### Statistical analysis

Overall survival was estimated by K–M analysis with log-rank test. The Wilcoxon rank-sum test was used to compare these measures between the groups. To compare three or more groups, a Kruskal-Wallis test was used. Correlation (Pearson correlation coefficient *r*) assessed strength and direction of the linear relationship between two variables. Statistical analyses and data visualization were performed in R programing language. *P*-values (*p*) < 0.05 were considered statistically significant.

## Results

### Identification of CC immune subtypes based on infiltration of immune cells

**Fig. 1:**
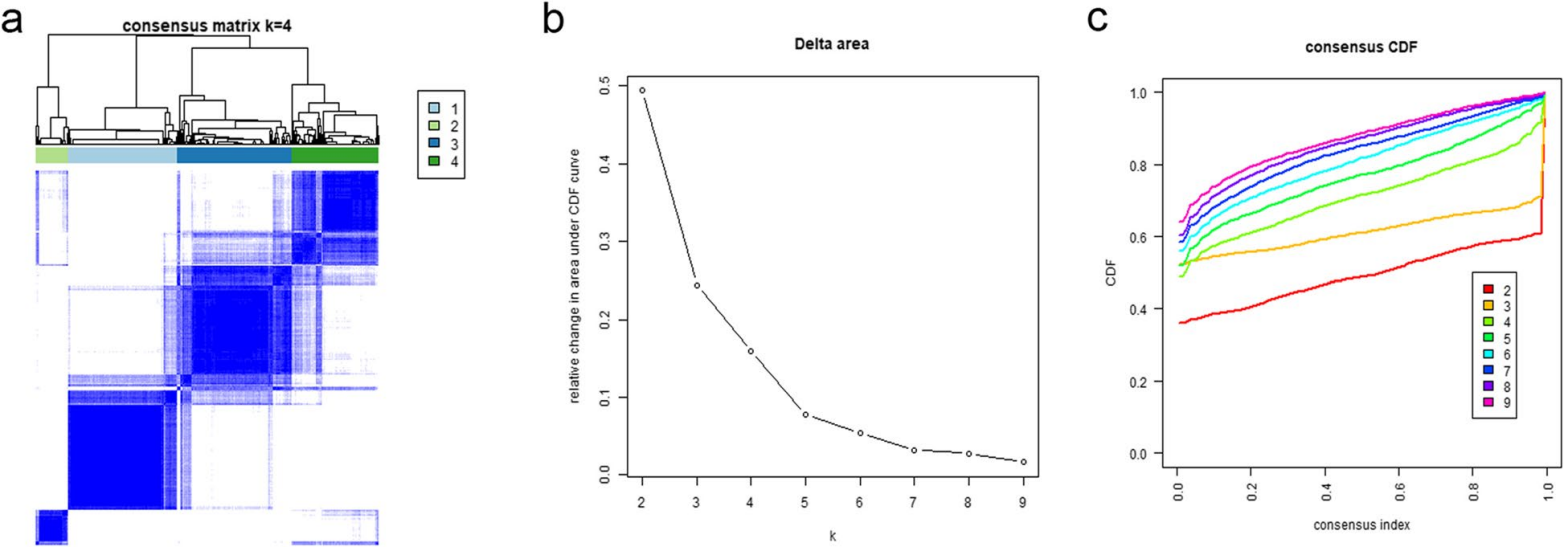
Consensus clustering of colon cancer (CC). **a** The TCGA samples were divided into four distinct clusters when *k* = 4. **b** Relative change in area under cumulative distribution function (CDF) curve for *k* = 2–9. **c** Consensus clustering CDF curve for *k* = 2–9

**Fig. 2:**
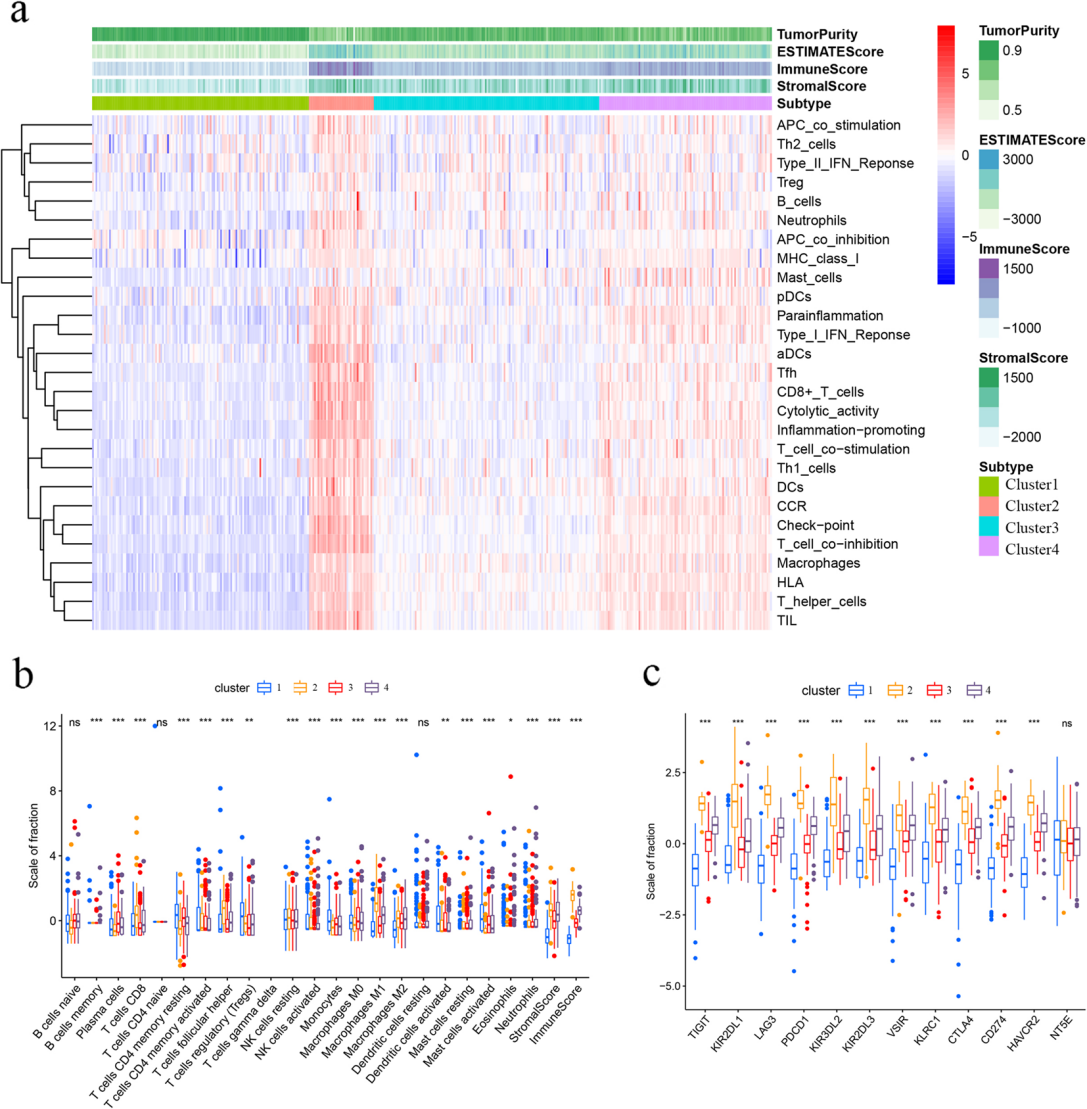
Determination of four immune subtypes in the CC. **a** Heatmap of ssGSEA scores. **b** The differences in the infiltrating immune cells between four clusters. **c** The differences in the expressions of immune checkpoint genes consisting among four subtypes. *P < 0.05, **P < 0.01, ***P < 0.005

Based on ssGSEA scores, the TCGA CC samples in this data set have been clustered into four immune infiltration subtypes: 127 cases in Cluster1, 38 cases in Cluster1, 132 cases in Cluster3, and 101 cases in Cluster4 (Figs. 1 and 2a). The CC cases with higher immune infiltration had higher ESTIMATE, stromal, and immune scores but a lower tumor purity among the 22 types of immune cells (*P* < 0.05). The infiltration of various types of immune cells showed a progressive increasing or decreasing trend, such as CD8 T cells, T cells follicular helper, NK cells resting, macrophages M1, and macrophages M2 have differences infiltration degree in different subtypes (*P* < 0.001) (Fig. 2b). Finally, the expression profiles of 12 immune checkpoint genes (ICIs) (i.e., LAG3, VSIR, CD274, KIR2DL1, KLRC1, HAVCR2, NT5E CTLA4, TIGIT, PDCD1, KIR3DL2, and KIR2DL3), which are crucial for immune modulation, were further examined. The expression correlated with high levels of immune infiltration cluster (Fig. 2c).

### Identification of DEGs

**Fig. 3:**
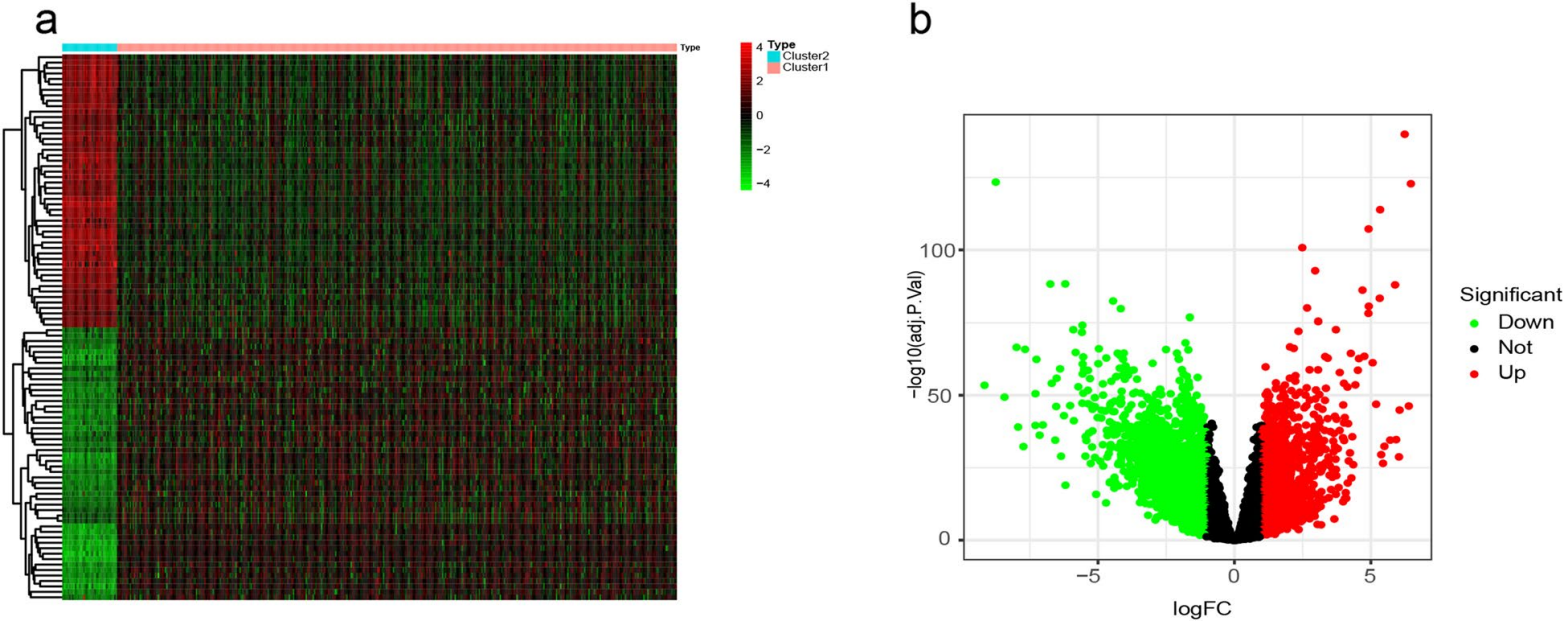
Analysis of differentially expressed genes. **a** Heatmap of differentially expressed genes. **b** The volcano graph shows the distribution of differential genes between Cluster1 and Cluster2, the red and blue dots represent significantly up- and downregulated expressed genes, respectively

In the TCGA dataset, we identified 3572 DEGs between the high- and low-infiltration subgroup (Cluster1 VS Cluster2) with the criteria of *P* < 0.05 and [logFC] > 1. Of the 3572 genes, 1343 genes were upregulated and 2229 downregulated. An expression volcano plots and heat map were indicated in Fig. 3a and b.

### Identification of co-expression modules

**Fig. 4:**
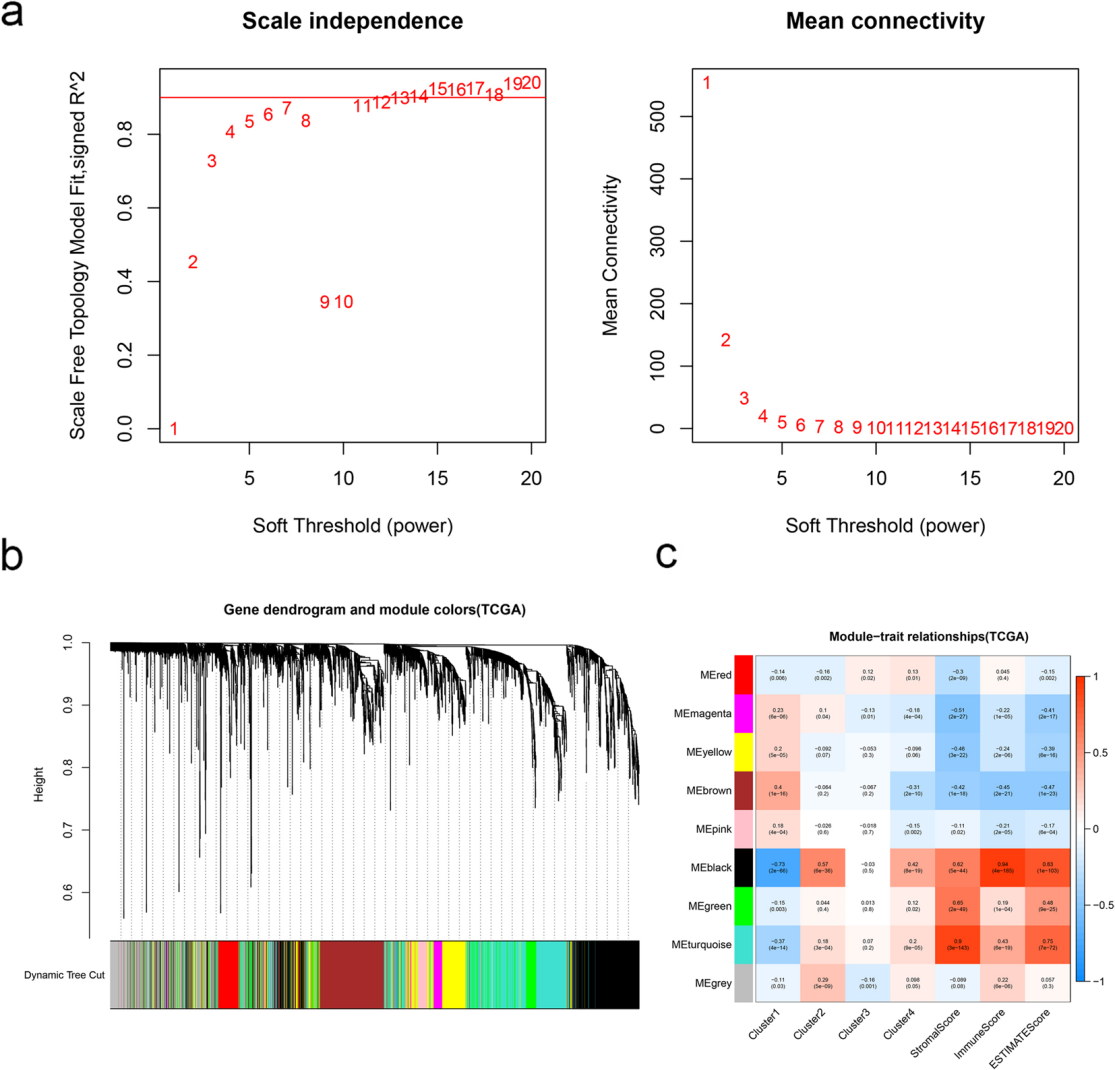
Weighted co-expression gene network. **a** Determination of the *β* parameter value in the adjacency function. **b** Nine gene modules were merged

**Fig. 5:**
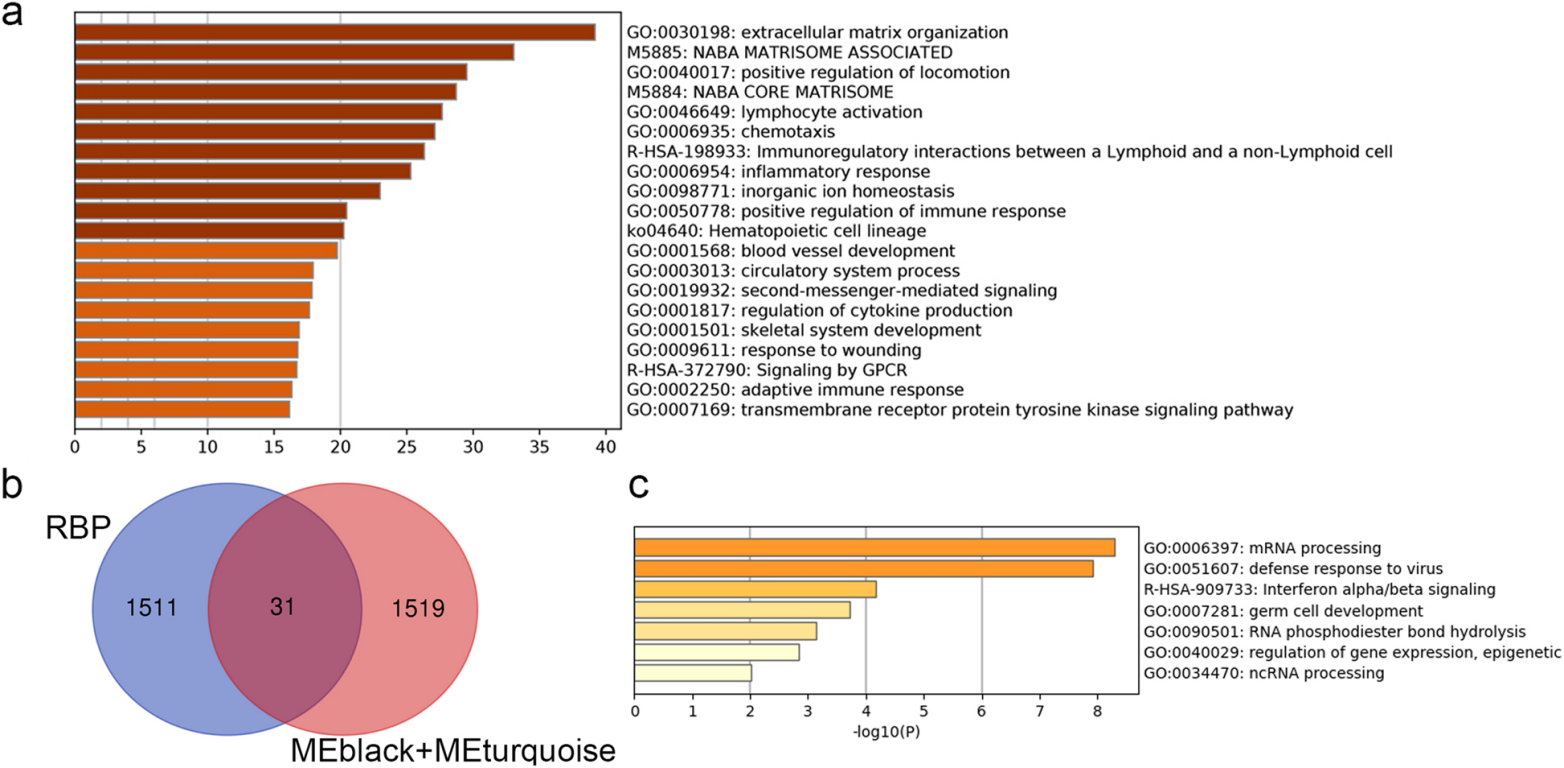
**a** GO function analysis of turquoise and black module genes. **b** Venn diagrams showing the intersection of two module genes and RBPs. **c** GO function analysis of thirty-one RBPs

We employed the WGCNA to analyze the DEGs. A soft threshold (power = 7) was selected by standard scale-free model fitting index R2 = 0.92 (Fig. 4a). Three modules shown in turquoise, green, and black in Fig. 4c were positively correlated with Cluster2, Cluster4, Stromal score, Immune score, and ESTIMATE score. Three modules shown in magenta, yellow, and brown negatively correlated with Cluster2, Cluster4, Stromal score, Immune score, and ESTIMATE score (Fig. 4c). Next, turquoise and black module were chosen to further analysis. Total 1550 genes from the two modules were applied to Gene Ontology (GO) analysis. The GO terms lymphocyte activation, immunoregulatory interactions between a lymphoid and a non-Lymphoid cell, inflammatory response, and positive regulation of immune response were the enriched GO terms (Fig. 5a). Then, we obtained 31 RBPs by taking the intersection of turquoise and black module genes and 1542 RBPs (Fig. 5b; Table 1). GO analysis revealed that the majority of these genes were functionally related to Mrna processing, defense response to virus, and interferon alpha/beta signaling (Fig. 5c; Table 2).

**Table 1:** The specific information of 31 RBPs

Gene Symbol	Description
SIDT1	SID1 transmembrane family member 1
A1CF	APOBEC1 complementation factor
IPO4	importin 4
DNMT3B	DNA methyltransferase 3 beta
QKI	QKI, KH domain containing RNA binding
PPARGC1A	PPARG coactivator 1 alpha
MEX3A	mex-3 RNA binding family member A
DZIP1	DAZ interacting zinc finger protein 1
TLR7	toll like receptor 7
RNASE1	ribonuclease A family member 1, pancreatic
POLR1G	RNA polymerase I subunit G
RBFOX3	RNA binding fox-1 homolog 3
DDX27	DEAD-box helicase 27
RBM24	RNA binding motif protein 24
PABPC3	poly(A) binding protein cytoplasmic 3
ZCCHC24	zinc finger CCHC-type containing 24
IFIT2	interferon induced protein with tetratricopeptide repeats 2
TLR8	toll like receptor 8
DDX60	DExD/H-box helicase 60
NPM2	nucleophosmin/nucleoplasmin 2
FBXO17	F-box protein 17
ZC3HAV1L	zinc finger CCCH-type containing, antiviral 1 like
DQX1	DEAQ-box RNA dependent ATPase 1
CELF3	CUGBP Elav-like family member 3
OASL	2'-5'-oligoadenylate synthetase like
CDK5RAP1	CDK5 regulatory subunit associated protein 1
PABPC1L	poly(A) binding protein cytoplasmic 1 like
TSEN2	tRNA splicing endonuclease subunit 2
IFIT1	interferon induced protein with tetratricopeptide repeats 1
AZGP1	alpha-2-glycoprotein 1, zinc-binding
RAVER2	ribonucleoprotein, PTB binding 2

**Table 2:** The biological processes of 31 RNA-binding proteins

Category	Term	Description
GO Biological Processes	GO:0006397	mRNA processing
GO Biological Processes	GO:0006397	mRNA processing
GO Biological Processes	GO:0008380	RNA splicing
GO Biological Processes	GO:0048024	regulation of mRNA splicing, via spliceosome
GO Biological Processes	GO:0050684	regulation of mRNA processing
GO Biological Processes	GO:0043484	regulation of RNA splicing
GO Biological Processes	GO:0000381	regulation of alternative mRNA splicing, via spliceosome
GO Biological Processes	GO:0000377	RNA splicing, via transesterification reactions with bulged adenosine as nucleophile
GO Biological Processes	GO:0000398	mRNA splicing, via spliceosome
GO Biological Processes	GO:0000375	RNA splicing, via transesterification reactions
GO Biological Processes	GO:0000380	alternative mRNA splicing, via spliceosome
GO Biological Processes	GO:1903311	regulation of mRNA metabolic process
GO Biological Processes	GO:0051607	defense response to virus
GO Biological Processes	GO:0051607	defense response to virus
GO Biological Processes	GO:0140546	defense response to symbiont
GO Biological Processes	GO:0009615	response to virus
GO Biological Processes	GO:0002221	pattern recognition receptor signaling pathway
Reactome Gene Sets	R-HSA-909733	Interferon alpha/beta signaling
Reactome Gene Sets	R-HSA-909733	Interferon alpha/beta signaling
GO Biological Processes	GO:0060337	type I interferon signaling pathway
GO Biological Processes	GO:0071357	cellular response to type I interferon
GO Biological Processes	GO:0034340	response to type I interferon
GO Biological Processes	GO:0043393	regulation of protein binding
Reactome Gene Sets	R-HSA-913531	Interferon Signaling
GO Biological Processes	GO:0051098	regulation of binding
GO Biological Processes	GO:0007281	germ cell development
GO Biological Processes	GO:0007281	germ cell development
GO Biological Processes	GO:0022412	cellular process involved in reproduction in multicellular organism
GO Biological Processes	GO:0010638	positive regulation of organelle organization
GO Biological Processes	GO:0007276	gamete generation
GO Biological Processes	GO:0090501	RNA phosphodiester bond hydrolysis
GO Biological Processes	GO:0090501	RNA phosphodiester bond hydrolysis
GO Biological Processes	GO:0090305	nucleic acid phosphodiester bond hydrolysis
GO Biological Processes	GO:0040029	regulation of gene expression, epigenetic
GO Biological Processes	GO:0040029	regulation of gene expression, epigenetic
GO Biological Processes	GO:0034470	ncRNA processing
GO Biological Processes	GO:0034470	ncRNA processing

### Construction of prognostic signatures based on RBP

**Fig. 6:**
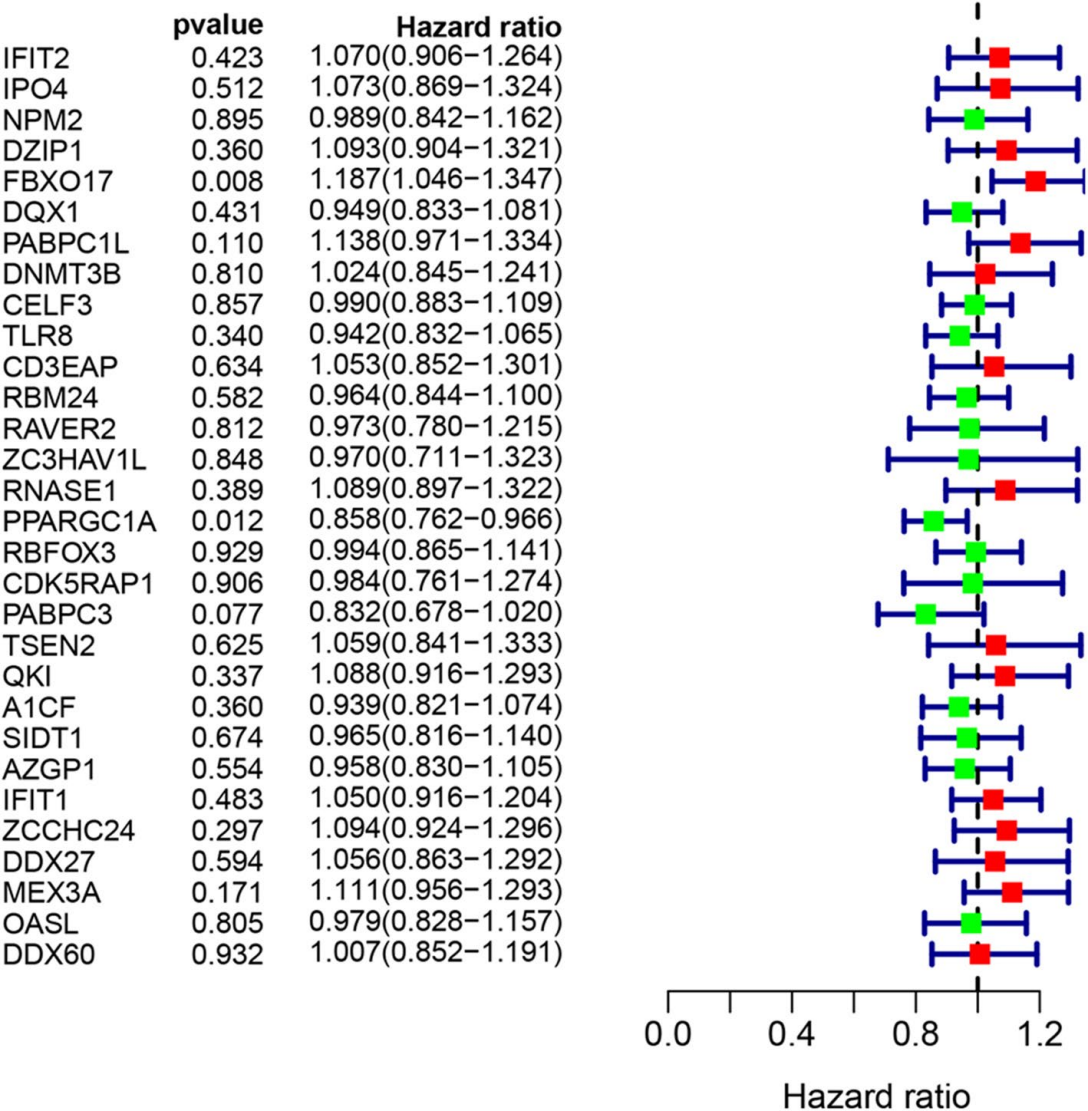
Screening of thirty-one RBPs related to significant prognosis in CC. Forest plot showing the prognostic RBPs

**Fig. 7:**
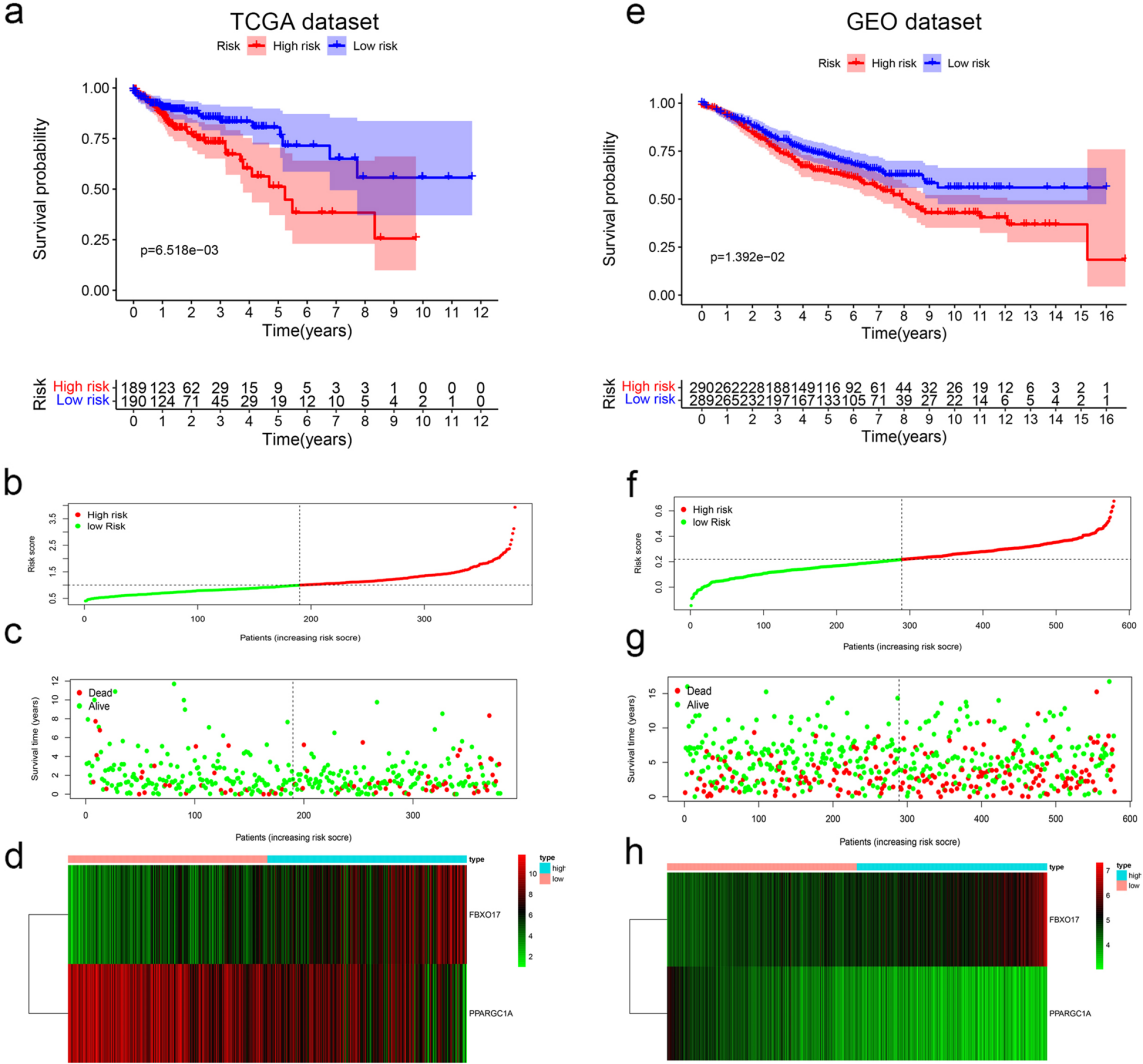
The RBP signature comprising two genes, which predicted the overall survival of CC, in the TCGA and GEO cohorts. **a** K-M survival analysis, **b** risk score distribution, **c** survival status, and **d** heatmap of a prognostic model in the CC cohort from TCGA. **e** K-M survival analysis, **f** risk score distribution, **g** survival status, and **h** heatmap of a prognostic model in the CC cohort from GEO

**Fig. 8:**
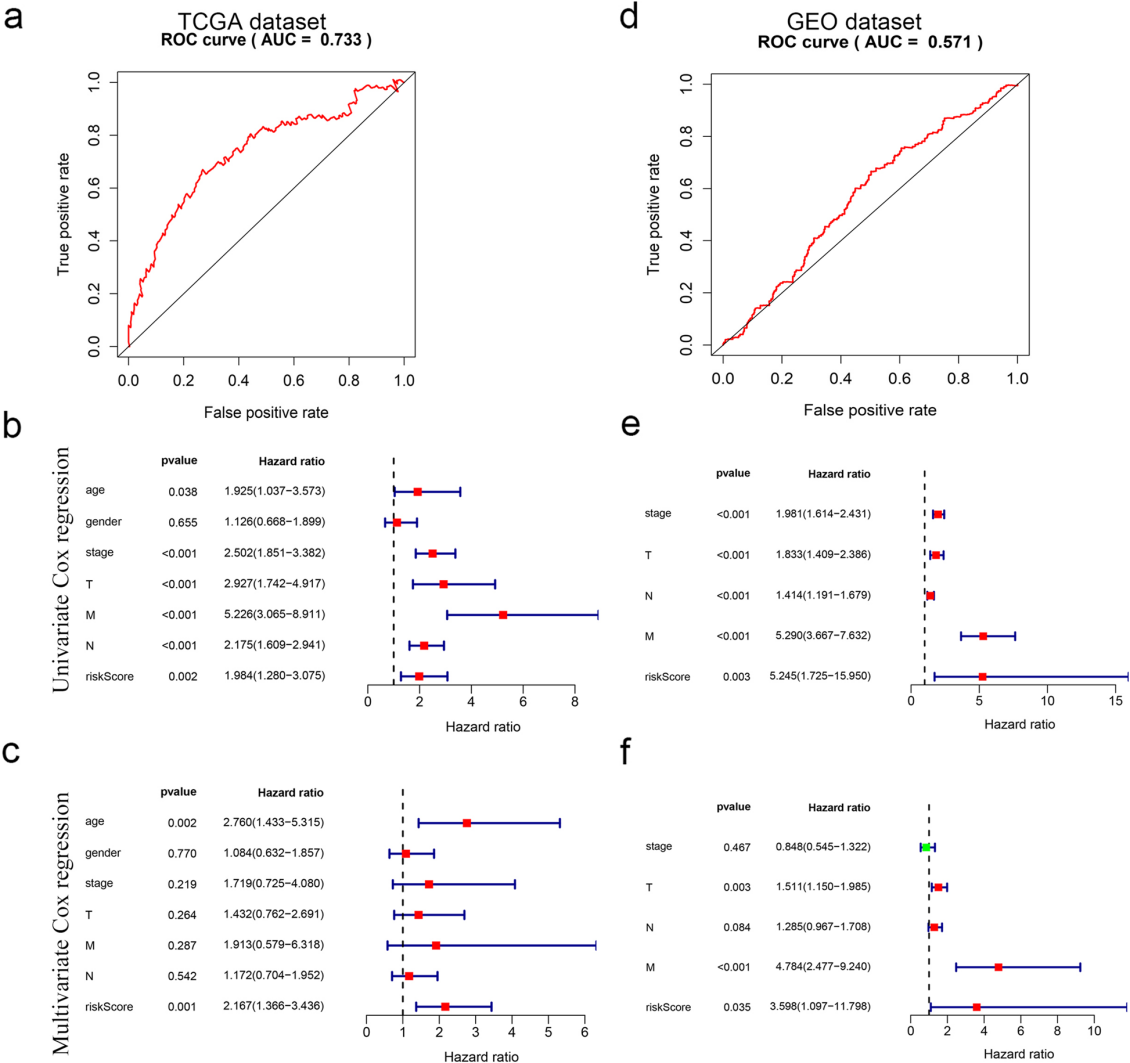
Prognostic value of two-gene signature in two datasets. **a** ROC curves, **b** UCR, and **c** MCR analysis of the risk score and other clinical indices in TCGA cohort. **d** ROC curves, **e** UCR, and **f** MCR, analysis of the risk score and other clinical indices in GEO cohort

From UCR analysis, we identified two candidate RBP genes associated with the prognosis in patients with CC (FBXO17 and PPARGC1A) (Fig. 6). Based on these findings, we established a CC prediction model by MCR analysis. We calculated the risk score for each patient by the following formula: Risk score = (0.1707 × Exp [FBXO17]) + (− 0.1515 × Exp [PPARGC1A]). Patients in the TCGA set were divided into high- or low-risk groups with the median risk score. K-M survival curves showed the OS was significantly worse in the high-risk group compared with the low-risk group (*P* = 6.518e− 03) (Fig. 7a). From the scatterplot and risk curve, the survival status of patients with different risk scores; the mortality rate remarkably increased with the higher risk score (Fig. 7b, c). Heatmap of expression profiles in TCGA indicated that FBXO17 were highly expressed in the high-risk group, but PPARGC1A were highly expressed in the low-risk group (Fig. 7d). Besides, the area under the ROC (AUC) values were 0.733 (Fig. 8a), showing a good capacity of two RBP genes in predicting overall survival. The prognostic significance of the prognostic mode was further validated by the validation cohort (Figs. 7 and 8e, h and d).

### Independent prognostic value of the risk model

We performed UCR analysis revealed that higher risk scores had shorter OS (HR, 1.984; 95% CI, 1.280−3.075; *P* = 0.002) (Fig. 8b). Several clinicopathologic variables displayed a relationship with prognosis included age, stage, metastasis, T, and N. Additional MCR analysis indicated that analyses indicated that high-level risk score served as an independent prognostic factor for poor survival in CC patients (HR, 2.167; CI, 1.366−2.346, *P* = 0.001) (Fig. 8c). To determine whether the clinical prognostic model was reliable, we then utilized this same risk score formula to analyze patients in the GEO cohort, which was consistent with those found in the TCGA database (Fig. 8e, f).

### Immune profile in risk groups

**Fig. 9:**
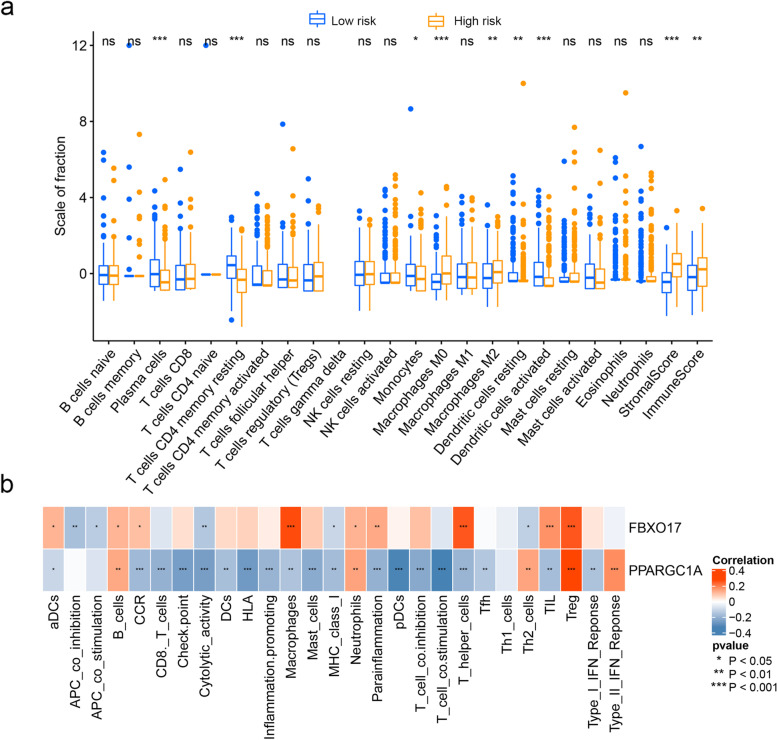
Analysis of the immune gene sets between different risk groups in the TCGA cohort. **a** The immune infiltration in high- and low-risk patients. **b** Correlation heatmap of two RBPs (FBXO17 and PPARGC1A) and immune gene sets. *P < 0.05, **P < 0.01, ***P < 0.005

We used the CIBERSORT algorithm to assess the composition of the immune microenvironment and further reveal the differences of immune cell infiltration between the two risk groups. As shown in Fig. 9a, plasma cells, T cells CD4 memory resting, monocytes, and dendritic cells activated cells were downregulated in the high-risk group, while Macrophages M0, Macrophages M2, Stromal score, and Immune score were significantly upregulated (*P* < 0.05) (Fig. 9a). Furthermore, PPARGC1A and FBXO17 expression negatively or positively associated with the immune gene sets, respectively (Fig. 9b).

### Drug sensitivity analysis of FBXO17 and PPARGC1A

**Fig. 10:**
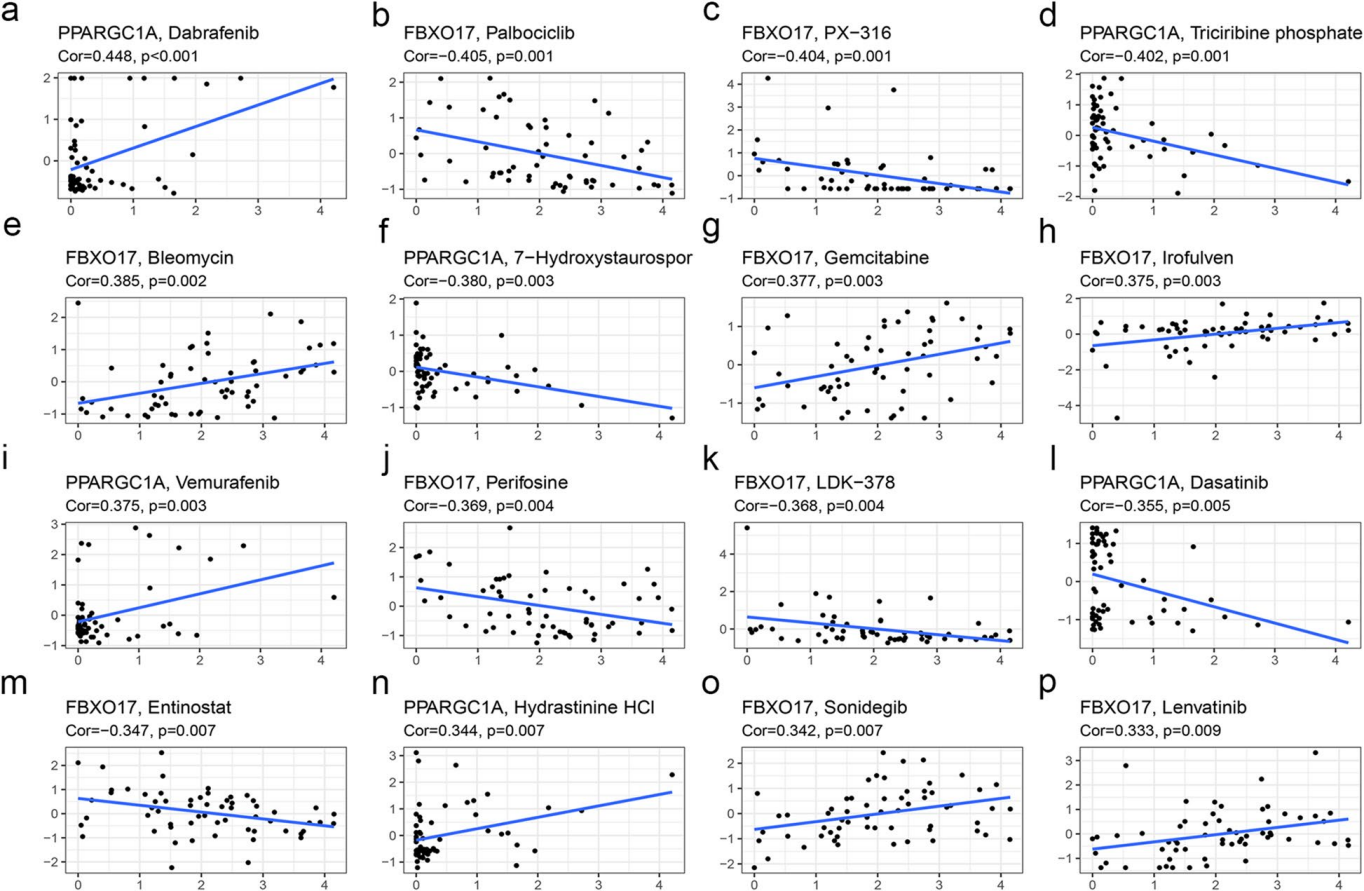
Drug sensitivity analysis of two RBPs (FBXO17 and PPARGC1A). FBXO17 expression was negatively associated with **b** Palbociclib, **c** PX-31, **j** Perifosine, **k** LDK-378, **m** Entinostat drug sensitivity, and positively related to **e** Bleomycin, **g** Gemcitabine, **h** Irofulven, **o** Sonidegib, and **p** Lenvatinib drug sensitivity. PPARGC1A expression was negatively related to **d** Triciribine phosphate, **f** 7-Hydroxystaurosporine, and **l** Dasatinib drug sensitivity, and positively related to **a** Dabrafenib, **i** Vemurafenib, and **n** Hydrastinine HCl drug sensitivity. *X*-axis is gene expression; *Y*-axis is IC50 of each drug

The CellMiner database was exploited to analyze the relationship between the drug sensitivity and the expression of FBXO17 and PPARGC1A. Pearson’s correlation analysis indicated that FBXO17 expression was negatively associated with Entinostat, LDK-378, Perifosine, PX-31, and Palbociclib drug sensitivity, and positively related to Bleomycin, Gemcitabine, Irofulven, Sonidegib, and Lenvatinib drug sensitivity. PPARGC1A expression was negatively related to Triciribine phosphate, 7-Hydroxystaurosporine, and Dasatinib drug sensitivity. PPARGC1A expression was positively related to Hydrastinine HCl, Vemurafenib, and Dabrafenib drug sensitivity (Fig. 10).

## Discussion

CC is one of the devastating malignancies with poor prognosis [22, 23]. Screening for candidate biomarkers remains a great challenge in improving the prognosis and evaluating the therapeutic effect in future. However, at present, no robust and effective biological markers can accurately predict the survival and prognosis of CC patients. It is well-accepted that immunity also plays critical roles in cancer development and progression [24]. Immunotherapy is showing impressive success in cancer treatment [25, 26]. Thus, it is significantly important to construct an immune-related prediction model, thus guiding the clinical treatment strategy.

Herein, CC patients were separated into four clusters based on the immune signature score. There are notable differences in tumor purity, ESTIMATE, stromal scores, immune scores, and the expression of immune checkpoint markers between the four clusters. The Cluster2 patients have a highest immune infiltration, and Cluster1 have a lowest immune infiltration. WGCNA analysis found turquoise and black module with highly relevant expression pattern. Then, the identified module genes, we performed Gene Ontology (GO) enrichment analysis in biological processes for “turquoise” and “black” module, to explore the biological process of each module. Two modules including 1550 DEGs were involved in lymphocyte activation, immunoregulatory interactions between a lymphoid and a non-lymphoid cell, inflammatory response, and positive regulation of immune response. Many studies have confirmed that there is a close relationship between tumor immune and CC [27]. Thirty-one IARBPs in two modules were selected out for further screening, analysis, and construction of a prognostic signature composed of two IARBPs.

Canonical RBPs work by binding to conserved sequence motifs in their target mRNAs via combinations of structurally well-defined RNA-binding domains (RBDs) [28]. Classic RBDs include the RNA recognition motif, the K-homology, DEAD/DEAH helicase, and zinc-finger domains [16]. Besides, non-canonical RBPs refer to those proteins which have not been proved to have classic RBDs or the established domains by direct experimental evidence but have RNA-binding activity. In our prognostic signature, the FBXO17 and PPARGC1A are classic RBPs. FBXO17 primarily plays an oncogenic role in various cancers [29, 30]. Over the past few years, studies have increasingly documented the contribution of FBXO17 to immune. For instance, FBXO17 inhibited activation of IFN-I signaling induced by various innate stimuli. Moreover, knockdown or knockout of FBXO17 promoted the transcriptional induction of IFN-β and IFN-stimulated genes as well as antiviral activities [31]. PPARGC1A is a known master regulator of mitochondrial biogenesis [32]. PPARGC1A variant appears to be associated with the risk of CC [33]. Additionally, PPARGC1A may directly affect expression of genes with either pro- or anti-inflammatory functions [34]. However, there are few studies on the role of PPARGC1A expression in tumor immunity. This needs further research in the future.

Next, the risk score was employed to divide patients into low- and high-risk group. K-M curve, score plot, and survival status plot, as well as ROC curve demonstrated that the two IARBPs had a favorable estimation potential in TCGA and GEO databases.

Furthermore, we researched on the relation of the two immune-associated RBPs prognostic signature to immune microenvironment. The high-risk group had significantly higher ESTIMATE score and immune score compared to low-risk group. For the type of infiltrating immune cells, the degree of infiltration of Macrophages M0 and Macrophages M2 were significantly upregulated. In the immune microenvironment of CC, tumor-associated macrophages (TAMs) are one of major tumor-infiltrating immune cells [35]. Although macrophages should be able to kill tumor cells, immunosuppressive microenvironment most often polarizes TAMs into M2-like macrophages rather than M1-like macrophages, which promote immunosuppression, angiogenesis, and extracellular matrix [36]. Many studies have reported that the presence of large numbers of M2 macrophages in colorectal carcinoma is significantly correlated with decreased survival rates [37, 38]. M2 macrophage infiltration is positively related with the expression of immune checkpoints. Furthermore, anti-M2 macrophages combined with immune checkpoint inhibitors improves the therapeutic effect and provides a new idea for the treatment of tumors [39]. Contrary to the pro-inflammatory function during infections, tumor-associated neutrophils (TANs) promotes tumor progression malignancy by mediating angiogenesis [40]. This indicated that high-risk patients might be in an immunosuppressive state and have a poor effect to the immunotherapy.

Furthermore, the CellMiner database was exploited to analyze the relationship between the drug sensitivity and the expression of FBXO17 and PPARGC1A. The result indicated that FBXO17 was most sensitive to treatment with Molecular targeted therapy drugs such as Irofulven, Sonidegib, and Lenvatinib. In addition, the PPARGC1A was most sensitive to Vemurafenib and Dabrafenib.

In conclusion, we performed a comprehensive bioinformatics analysis of RBPs and identified a novel prognostic risk score involving two RBPs in CC. This risk model can be used as a biomarker to independently predict the prognosis of CC and may offer new promising therapeutic targets for CC patients. The limitation of this study is that further research is still needed to verify our findings.

## Supplementary Information

**Additional file 1: Figure S1.** The work-flow of this study.

## Data Availability

The datasets analyzed were acquired from The Cancer Genome Atlas (TCGA) database (https://portal.gdc.cancer.gov/) and GEO database (http://www.ncbi.nlm.nih.gov/geo/).

## Abbreviations

TCGA: The Cancer Genome Atlas databases
GEO: Gene Expression Omnibus
CC: Colon cancer
WGCNA: Co-expression network analysis
RBPs: RNA-binding proteins
IARBPs: Immune-associated RNA-binding proteins
DEGs: Differentially expressed genes
GO: Gene Ontology
UCR: Univariate Cox regression
MCR: Multivariate Cox regression
ssGSEA: Single sample gene set enrichment analysis
