# Supplementary figures and images for: Identification and validation of an immune-associated RNA-binding proteins signature to predict clinical outcomes and therapeutic responses in colon cancer patients
Source: World J Surg Oncol. 2021 Oct 26;19:314. doi: 10.1186/s12957-021-02411-2 (PMC8549210; doi:10.1186/s12957-021-02411-2)

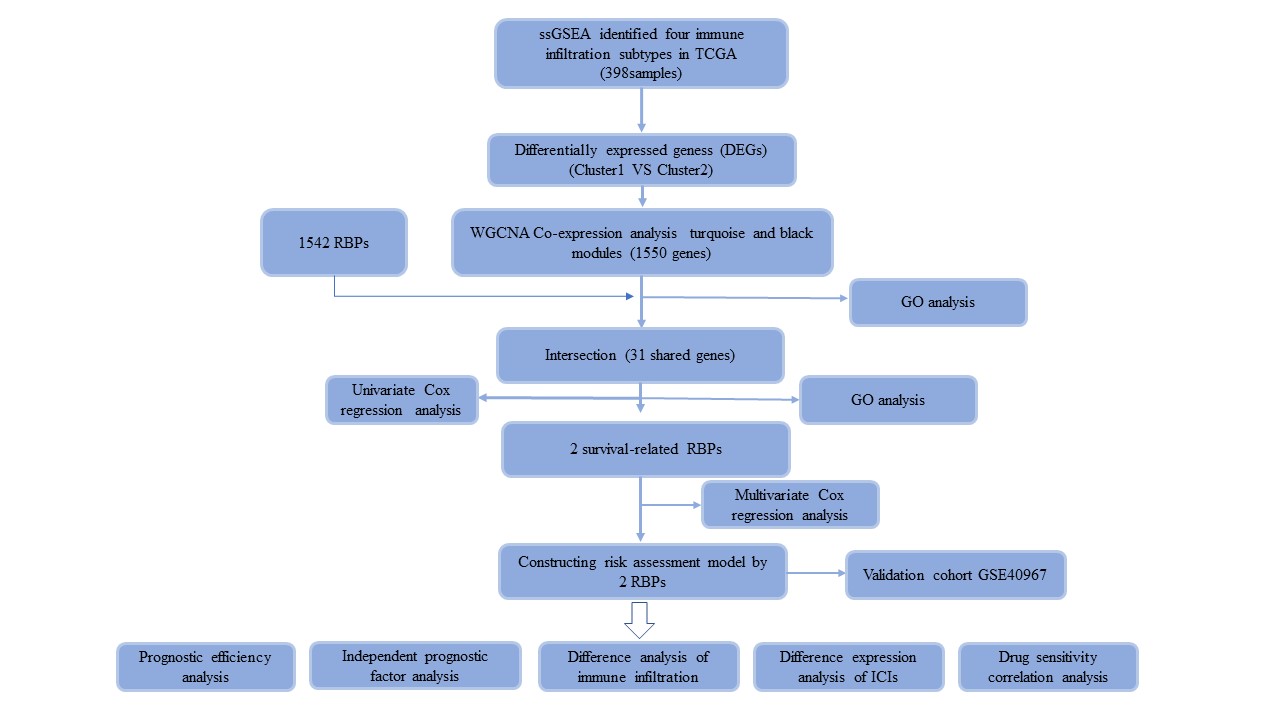

Supplement: Supplementary file 1 — Additional file 1: Figure S1. The work-flow of this study. [file 12957_2021_2411_MOESM1_ESM.jpg]
